# Serum Uric Acid Levels at Admission Could Predict the Chronic Post-stroke Fatigue

**DOI:** 10.3389/fnut.2022.850355

**Published:** 2022-02-22

**Authors:** Wenwei Ren, Junxin Wu, Zijing Wu, Shuang Yang, Xiaofang Jiang, Minjie Xu, Beilan Wu, Caixia Xie, Jincai He, Xin Yu

**Affiliations:** ^1^School of Mental Health, Wenzhou Medical University, Wenzhou, China; ^2^Department of Neurology, The First Affiliated Hospital of Wenzhou Medical University, Wenzhou, China; ^3^Peking University Institute of Mental Health (Sixth Hospital), Beijing, China; ^4^National Clinical Research Center for Mental Disorders and Key Laboratory of Mental Health, Ministry of Health, Peking University, Beijing, China; ^5^Beijing Municipal Key Laboratory for Translational Research on Diagnosis and Treatment of Dementia, Beijing, China

**Keywords:** stroke, fatigue, uric acid, risk, association

## Abstract

**Background:**

Post-stroke fatigue (PSF) is a frequent complication of stroke. Serum uric acid (SUA) is frequently thought to be a risk factor for stroke. This study aimed to investigate whether SUA also played a role in PSF.

**Methods:**

Subjects with ischemic stroke were screened from The First Affiliated Hospital of Wenzhou Medical University between January 2020 and October 2020. Patients' fatigue symptoms were assessed by the Fatigue severity scale (FSS). To investigate the relationship between SUA and PSF, binary logistic regression analysis was conducted, with the confounders being controlled. SUA levels were divided into four layers (Q1 ≤ 245 μmol/L; Q2 246–308 μmol/L; Q3 309–365 μmol/L; Q4 ≥366 μmol/L) based on the quartiles.

**Results:**

SUA levels were significantly higher in the PSF group (345.96 ± 73.78 μmol/L) than the non-PSF group (295.97 ± 87.8 μmol/L, *P* < 0.001). There were no differences in any other variables between these two groups. After adjusting the confounders, the risk of PSF in the Q4 layer (≥366 μmol/L) was 6.05 times (95% CI 1.79–20.43, *P* = 0.004) higher than that in Q1 (≤245 μmol/L).

**Conclusion:**

High SUA at admission was an independent risk factor for fatigue 1 year after stroke onset. High SUA (≥366 μmol/L) during stroke deserves more attention, and active control of high SUA levels may be beneficial to reduce the incidence of PSF in the chronic stage following stroke.

## Introduction

PSF is a common complication after stroke and predominantly presents lacking energy, excessive sleepiness, fatigability after activity, napping, or increased stress-induced sensitivity (1). According to previous studies, the incidence of PSF ranged from 25–85%, with a pooled prevalence estimated at 50% (95% CI: 43–57%) (2). More than half of patients with mild stroke who had little or no residual neurological deficits suffered from PSF (3). PSF was the second major barrier to the recovery of stroke survivors (4). Accumulating studies had observed that fatigue could lead to poorer quality of life, and higher rates of disability and mortality (5–10). Previous studies had demonstrated that fatigue and depressive symptoms were highly correlated (5, 11), and 34–49% of patients with fatigue were accompanied by depression symptoms (12, 13). It should be noted that fatigue also occurred in 47% of non-depressed stroke survivors (2). Previous studies on PSF often failed to exclude the confusion caused by depression (14–17). This study will focus on fatigue patients without depression symptoms, which can reduce the confounding effects caused by depression (11).

Uric acid is a metabolite of purine degradation, which can lead to kidney stones and gout (18). The biological functions of UA are bilateral. On one hand, it has been discovered that uric acid has an antioxidant property (19, 20), which contributes to nearly 50% of human plasma's antioxidant capacity (21). Accumulating studies had found its protective effect in different neurologic diseases, such as Parkinson's disease, amyotrophic lateral sclerosis, and multiple sclerosis (22–25). On the other hand, uric acid may also have pro-inflammatory effects on vascular cells and could generate radicals through the stimulation of NADPH oxidase (26–28).

Post-stroke fatigue could be classified as early and late fatigue, and early fatigue was defined as within 3 months post-stroke (acute stage) and late as more than 3 months post-stroke (chronic stage) (29, 30). In addition, previous studies had found that fatigue prevalence may decrease in the acute stage (1–3 months post-stroke), and then keep stable in the chronic stage (over 3 months) (31, 32). It should be noted that fatigue occurring in the acute stage following stroke was considered as a general non-specific response to the major disruptive event caused by stroke, while, fatigue in the chronic stage following stroke was considered to be a stroke-related sequel (33). A previous study observed that low SUA levels at admission were related to an increased risk of fatigue during the acute stage of stroke (17). As PSF occurred in the acute stage was considered to be a general non-specific response instead of a stroke-related sequel, the purpose of this study was to see if SUA at admission was also related to PSF in the chronic stage of stroke.

## Methods

### Participants

Patients with acute ischemic stroke were screened between January 2020 and October 2020, from the First Affiliated Hospital of Wenzhou Medical University. Inclusion criteria were listed below: (1) age ≥ 18 years old; (2) diagnosed with acute ischemic stroke, and confirmed by magnetic resonance imaging; Exclusion criteria were listed below: (1) with a history of psychiatric disorder; (2) with diseases related to fatigue including cancer, multiple sclerosis, Parkinson's disease, and systemic lupus erythematosus; (3) declined to participate; (4) Patient Health Questionnaire-9 (PHQ-9) score ≥ 5; (5) data incomplete.

**Figure 1:**
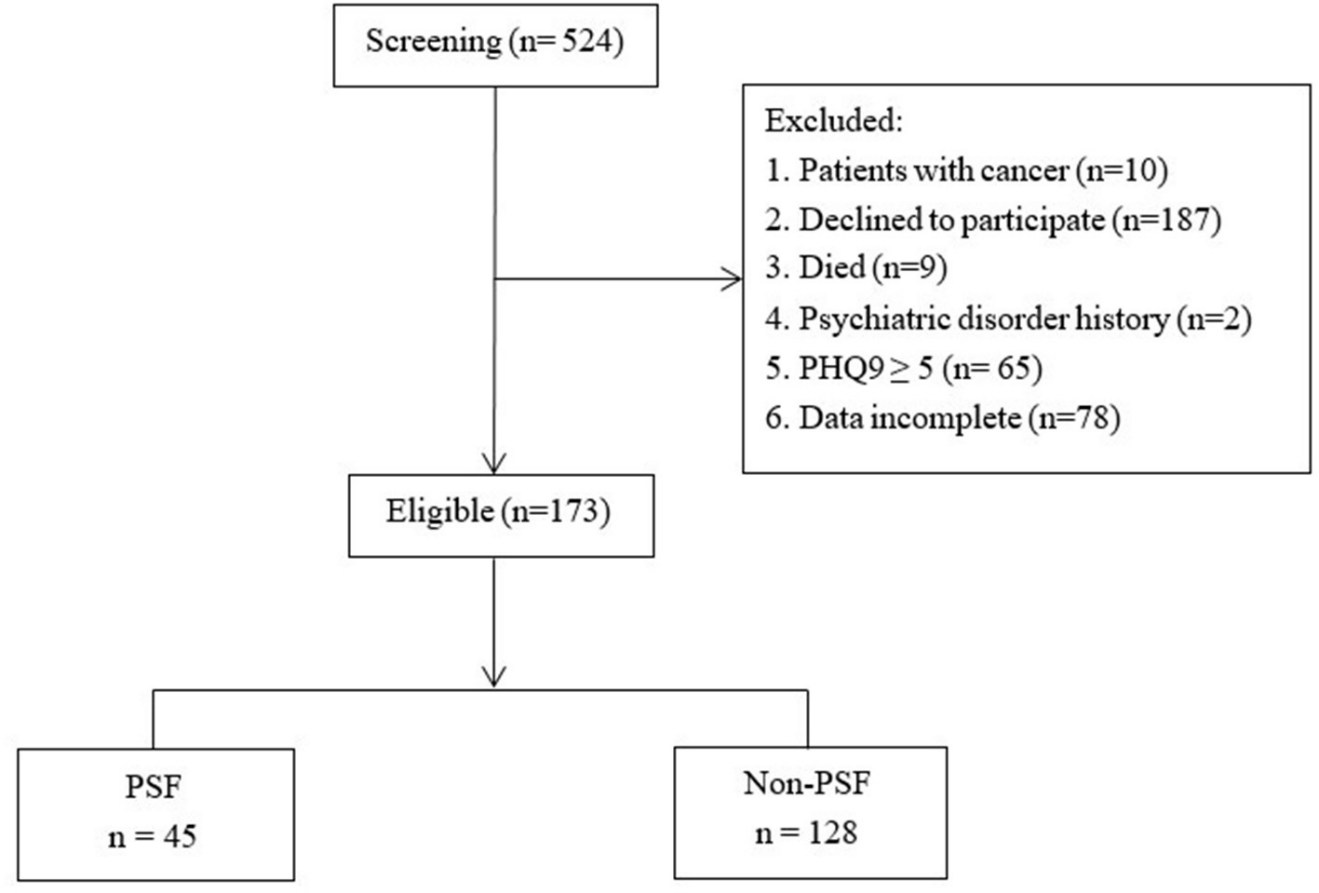
The flow of participants. PSF, Post-stroke fatigue; PHQ-9, Patient Health Questionnaire-9.

As was shown in Figure 1, 524 patients were screened, of which 351 were excluded and 173 patients were finally recruited.

The Ethics Committee of the First Affiliated Hospital of Wenzhou Medical University had approved this study. Due to the overall impact of COVID-19, all the participants provided verbal informed consent and finished the questionnaires by telephone.

### Data Collection

The baseline characteristics were obtained from the electronic clinical records. General demographic information included age, gender, smoking history, drinking history, and Body Mass Index (BMI). Comorbidity data included hypertension, diabetes, and atrial fibrillation. Clinical data included systolic and diastolic blood pressure, length of hospital stay, National Institutes of Health Stroke Scale (NIHSS) score, and stroke etiology (TOAST criteria). No patients had used anti-uric acid medication at admission. Blood samples were obtained in the morning, after 12 h of fasting. Laboratory data including SUA and creatinine were measured in the hospital biochemistry department. Functional outcomes were evaluated by telephone 1-year post-stroke by the modified Rankin Scale (mRS) and the Barthel Index (BI).

### Clinical Assessments

#### Depression

Depression was assessed by the PHQ-9, a nine-item questionnaire for depression screening. Each item of the scale is scored from “0” (not at all) to “3” (nearly every day). Participants with the PHQ-9 score ≥ 5 were considered to be depressed and were excluded in this study to eliminate the effects of depression on fatigue (34).

#### Fatigue

Fatigue was assessed by the Fatigue Severity Scale (FSS), a nine-item questionnaire for fatigue screening. Each item of the scale is scored from “1” (strongly disagree) to “7” (strongly agree), and a higher average score indicates a higher fatigue level. An average score of 4 or above is indicative of fatigue (35).

#### Statistical Analysis

Continuous data were displayed as mean ± standard deviation (mean ± SD) or median (inter-quartile range) based on the distribution of the data, and Student's *t*-test or Mann-Whitney U test was adopted separately to analyze the differences between the PSF and non-PSF groups. Categorical variables were shown as frequencies or percentages, which were analyzed by the Chi-square test or the Fisher's exact test. Binary logistic regression analysis was used to explore the potential predictors of PSF, and the results were displayed by the forest plot. Except for the SUA, age, gender, creatinine, mRS, and stroke etiology (TOAST criteria) which can influence the SUA and fatigue levels were also included in the regression model, and SUA levels were divided into four layers (Q1 ≤ 245 μmol/L; Q2 246–308 μmol/L; Q3 309–365 μmol/L; Q4 ≥366 μmol/L) based on the quartiles, with the Q1 being the reference.

SPSS 16.0 was used to conduct all the statistical analyses, and *P* < 0.05 was suggestive of statistical significance.

## Results

### Characteristics Between the PSF and Non-PSF Groups

Table 1 had demonstrated the demographic and clinical characteristics between the PSF and non-PSF groups. The SUA levels were significantly higher in the PSF group (345.96 ± 73.78 μmol/L) than the non-PSF group (295.97 ± 87.80 μmol/L, *P* < 0.001). There were no differences between the two groups in any other variables (all *P* > 0.05).

**Table 1:** Comparison of characteristics between the PSF and Non-PSF groups.

**Variables**	**Non-PSF**	**PSF**	**χ2/t/U**	***P*** **-value**
	**(*n* = 128)**	**(*n* = 45)**		
Age, Mean ± SD	53.95 ± 8.85	54.67 ± 7.82	−0.508	0.612
Gender(male), *n* (%)	104 (81.25)	38 (84.44)	0.065	0.799
BMI, Mean ± SD	24.79 ± 2.98	24.7 ± 2.86	0.192	0.848
Hypertension, *n* (%)	78 (60.94)	29 (64.44)	0.057	0.812
Heart disease, *n* (%)	5 (3.91)	2 (4.44)	Fisher	1
Diabetes, *n* (%)	39 (30.47)	11 (24.44)	0.331	0.565
Atrial fibrillation, *n* (%)	7 (5.47)	2 (4.44)	Fisher	1
Smoking, *n* (%)	75 (58.59)	21 (46.67)	1.465	0.226
Drinking, *n* (%)	55 (42.97)	16 (35.56)	0.481	0.488
SBP (mmHg), Mean ± SD	151.67 ± 25.84	157 ± 23.41	−1.278	0.205
DBP (mmHg), Mean ± SD	90.39 ± 16.57	93.82 ± 14.39	−1.321	0.19
Length of hospital stay, Median (IQR)	8 (7, 10)	7 (6, 9)	3284.5	0.159
**TOAST**, ***n*** **(%)**				
Large artery atherosclerosis	66 (51.56)	29 (64.44)	1.742	0.187
Non-large artery atherosclerosis	62 (48.44)	16 (35.56)		
NIHSS, Median (IQR)	1 (0, 2)	1 (0, 2)	2826.5	0.85
PHQ-9, Median (IQR)	1 (0, 2)	1 (0, 2)	2835.5	0.874
mRS, Median (IQR)	1 (0, 1)	0 (0, 1)	3230	0.186
Barthel Index, Median (IQR)	100 (100, 100)	100 (100, 100)	2956.5	0.55
SUA, Mean ± SD	295.97 ± 87.80	345.96 ± 73.78	−3.714	<0.001^***^

*** *P < 0.001*.

*PSF, Post-stroke fatigue; BMI, body mass index; SBP, Systolic blood pressure; DBP, Diastolic blood pressure; TOAST, stroke classification criteria; NIHSS, National Institutes of Health Stroke Scale; PHQ-9, Patient Health Questionnaire-9; mRS, modified Rankin Scale; SUA, serum unic acid; SD, standard deviation; IQR, interquartile range; Fisher, Fisher exact test*.

### SUA and PSF

**Figure 2:**
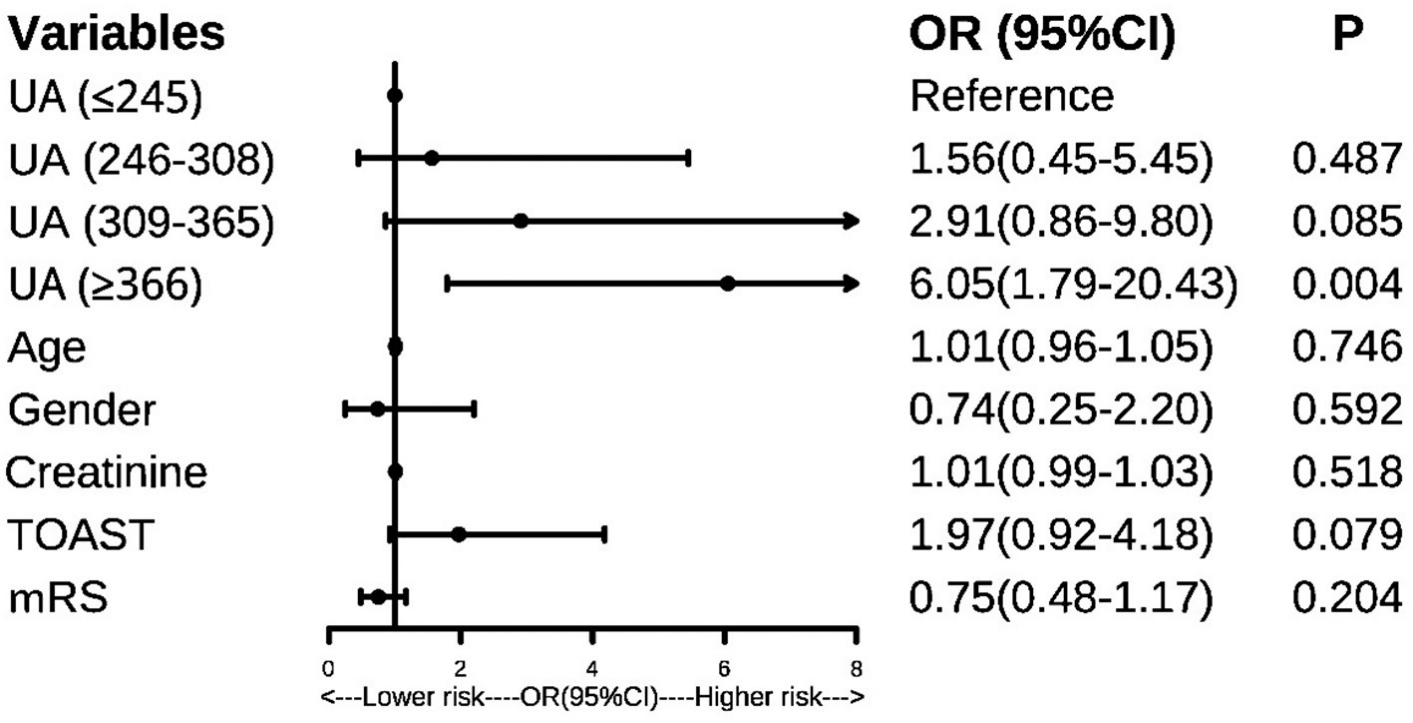
Forest plot, predictors of post-stroke fatigue. UA, uric acid; mRS, modified Rankin Scale; TOAST, stroke classification criteria.

The binary logistic regression analysis showed that patients in the Q4 layer showed a higher risk of PSF than those in Q1 (OR:5.47, 95%CI: 1.80–16.60, *P* = 0.003). As was shown in Figure 2, after controlling the confounders, patients in the Q4 layer still had a 6.05–fold increased risk of PSF than those in Q1. The results remained unchanged when the SUA levels were taken as continuous variables in the model (OR:1.008, 95% CI: 1.003–1.013, *P* = 0.003).

## Discussion

To our knowledge, this study is the first to investigate the relationship between SUA and PSF in the chronic stage following stroke. In this study, a high SUA level was a risk factor of fatigue 1 year after stroke onset. The prevalence of PSF was 6.05 times higher in the high SUA group (≥366 μmol/L) than in the low SUA group (≤245 μmol/L).

The relationship between SUA and stroke had been widely studied. SUA was found to be an independent risk factor for ischemic stroke (36) and non-fatal stroke (37), furthermore, SUA could predict poor outcomes of stroke (38). A recent meta-analysis including 15 prospective studies has shown that higher SUA levels indicated a higher incidence of stroke and risk of mortality of stroke (39). In addition, high SUA levels were also associated with post-stroke cognitive impairment and post-stroke depression (40–42). The relationship between SUA and PSF was rarely studied. A previous cross-sectional study by Shang et al. observed that a lower SUA level may indicate a higher risk of PSF during the acute stage of stroke (17), which was inconsistent with our study. Several reasons may explain this difference. On one hand, fatigue was assessed during the acute stage in the study by Wu et al. (17). While fatigue in this study was assessed 1 year after stroke (chronic stage). On the other hand, 82.6% of patients in the fatigue group had depression symptoms in the study by Shang et al. However, SUA was also found to be related to post-stroke depression (40). Therefore, in this study, patients with depression symptoms were excluded, which may reduce the confounding effects caused by depression to some extent. It was worth noting that another study in humans involving 54 healthy volunteers also found that increased SUA levels were associated with fatigue (43), which was consistent with our study. In addition, studies in animals found that fatigue after partial hepatectomy in rats was associated with increased hypoxanthine and UA levels (44). Besides, It was also observed in horses that plasma inosine Monophosphate concentration increased, ATP concentration decreased, and UA concentration significantly increased after a short period of high-intensity exercise (45). Given above, SUA may play an important role in fatigue. The mechanism underlying the SUA and fatigue remained unclear. It was inferred that systemic inflammatory response may play a significant role in the relationship between SUA and PSF. It had been studied that SUA was associated with inflammatory markers including TNF-alpha, IL-6, IL-1ra, IL-18, neutrophils count, and C-reactive protein (CRP) (46). It was worth mentioning that IL-6, TNF-alpha, IL-1β were all significantly associated with fatigue (47–49). Besides, a study in patients with breast cancer found that CRP levels significantly decreased with the improvement of fatigue (50). Another study also found that PSF was associated with higher levels of CRP (51). Furthermore, a recent study found that UA could activate the pyrin domain containing 3 (NLRP3) inflammasome. After the formation of the NLRP3 inflammasome, NLRP3 inflammasome-dependent caspase-1 activation could stimulate macrophages to secrete interleukin-1β (IL-1β) (28). It had been found that interleukin-1β (IL-1β) was positively correlated with the PSF at 6 months after stroke (52). Given above, systemic inflammation may play an important role between SUA and PSF (29).

The limitations of this study are listed as follows. 1. This study only measured SUA levels once at admission. As SUA levels may fluctuate over time, it is best to test SUA more times in the future; 2. The sample size is relatively small, which should be further expanded in the future; 3. Since it is a retrospective study, the causal relationship between SUA and PSF cannot be drawn. Future intervention studies are needed to further analyze the relationship between SUA and PSF.

## Conclusion

High SUA level at admission was an independent risk factor for fatigue 1 year after stroke onset. Patients with high SUA levels at admission deserve more attention, and active control of SUA levels may be beneficial for reducing the incidence of PSF in the chronic stage following stroke.

## Data Availability Statement

The raw data supporting the conclusions of this article will be made available by the authors, without undue reservation.

## Ethics Statement

The studies involving human participants were reviewed and approved by the Ethics Committee of the First Affiliated Hospital of Wenzhou Medical University. Written informed consent for participation was not required for this study in accordance with the national legislation and the institutional requirements.

## Author Contributions

XY, JH, and WR engaged in the study's conception and design. WR was responsible for the data statistics and paper writing. XY and JH reviewed the manuscript. All the following authors WR, JW, ZW, SY, XJ, MX, BW, and CX were responsible for collecting the data. All authors contributed to the article and approved the submitted version.

## Funding

This work was supported in part by the National Key R&D Programme of China (2018YFC1314200).

## Conflict of Interest

The authors declare that the research was conducted in the absence of any commercial or financial relationships that could be construed as a potential conflict of interest.

## Publisher's Note

All claims expressed in this article are solely those of the authors and do not necessarily represent those of their affiliated organizations, or those of the publisher, the editors and the reviewers. Any product that may be evaluated in this article, or claim that may be made by its manufacturer, is not guaranteed or endorsed by the publisher.
